# The Efficacy of MRI-Based ADC Measurements in Detecting Axillary Lymph Node Metastasis: Evaluation of a Prospective Study

**DOI:** 10.3390/curroncol31110487

**Published:** 2024-10-24

**Authors:** Faruk Türkeş, Özcan Dere, Funda Dinç, Cenk Yazkan, Önder Özcan, Okay Nazlı

**Affiliations:** 1 Özel Yücelen Hastanesi, Muğla 48600, Turkey; farukturkes@mu.edu.tr; 2 Department of General Surgery, Faculty of Medicine, Muğla Sıtkı Koçman University, Muğla 48121, Turkey; cenkyazkan@mu.edu.tr (C.Y.); onderozcan@mu.edu.tr (Ö.Ö.); okaynazli@mu.edu.tr (O.N.); 3 Department of Radiology, Faculty of Medicine, Muğla Sıtkı Koçman University, Muğla 48000, Turkey; fudadinc@mu.edu.tr

**Keywords:** axillary lymph node metastasis, apparent diffusion coefficient (ADC), breast cancer, sentinel lymph node biopsy (SLNB)

## Abstract

**Objective:** This study aimed to evaluate the efficacy of MRI-based Apparent Diffusion Coefficient (ADC) measurements in detecting axillary lymph node metastasis in breast cancer patients. By comparing preoperative MRI findings with intraoperative sentinel lymph node biopsy (SLNB) and postoperative pathological results, we sought to explore the potential of ADC values as a non-invasive alternative to axillary interventions. **Methods:** A total of 104 female patients diagnosed with breast cancer between 2019 and 2021 were included in this prospective study. ADC values of axillary lymph nodes, tumors, and muscle tissues were measured using a 3T MRI system. The correlation between these measurements and pathological outcomes was analyzed. Statistical analyses, including *t*-tests, ANOVA, and ROC curve analysis, were employed to assess the diagnostic performance of ADC values. **Results:** The results indicated that, while the mean ADC values of metastatic lymph nodes were lower than those of benign nodes, the sensitivity and specificity of MRI-based ADC measurements were inferior to the expected standards. The tumor ADC value and the tumor-to-lymph node ADC ratio were found to be more reliable indicators of metastasis than the lymph node ADC value alone. The diagnostic power of the tumor ADC value was significant, with a sensitivity of 75% and a specificity of 73%. **Conclusions:** MRI-based ADC measurements, particularly the tumor ADC value and the tumor-to-lymph node ADC ratio, show promise as potential non-invasive markers for axillary lymph node metastasis in breast cancer patients. However, the current results suggest that ADC measurements cannot yet replace SLNB in clinical practice.

## 1. Introduction

Breast cancer is the most common cancer among women, and the prognosis is determined by the stage at diagnosis. Axillary staging and axillary interventions are the sine qua non of the disease. In surgical procedures performed for axillary staging, malignant disease is not detected in up to 70% of cases [1,2]. In a recent meta-analysis, it was observed that the pathological complete response in axillary interventions varied between 15% and 62%, depending on the tumor subtype [3]. However, many complications, such as arm pain, lymphedema, and seroma, are encountered [4,5]. Magnetic resonance imaging (MRI) and ultrasound (US) can be used to non-invasively assess axillary lymph nodes, but they have not replaced pathological evaluation due to several limitations such as cortical thickness, fatty hilum, enhancement patterns, and lymph node grouping [6,7,8]. The SOUND randomized clinical trial did not recommend sentinel lymph node procedures in breast cancer patients with tumor sizes less than 2 cm, where no pathological lymph nodes were detected by ultrasound [9]. This evolution over time is now moving towards the complete elimination of axillary interventions in early-stage patients. In this study, we investigated the role of the apparent diffusion coefficient (ADC) value measured on MRI and lymph node cortex thickness in preventing axillary interventions.

## 2. Materials and Methods

This study was conducted in the Department of General Surgery at Muğla Sıtkı Koçman University, with the approval of the Clinical Research Ethics Committee of Muğla Sıtkı Koçman University Faculty of Medicine (decision dated 28 November 2019, No. 17/III). A total of 104 patients who were diagnosed with breast cancer, were operated on at our center, and did not meet any exclusion criteria were prospectively and consecutively included in this study. All patients provided written informed consent after being informed about this study.

Patients who had undergone breast US, mammography (MMG), and breast MRI at the time of diagnosis and were scheduled for procedures such as breast-conserving surgery (BCS), mastectomy, sentinel lymph node biopsy (SLNB), or axillary lymph node dissection (ALND) between 2019 and 2021 were included in this study. Male patients diagnosed with breast malignancy were excluded.

During this study, the demographic, radiological, and pathological data of the patients were evaluated. The parameters assessed included breast localization, multifocality and multicentricity, lesion BI-RADS classification (American College of Radiology Breast Imaging Reporting and Data System Atlas 5th edition), tumor size, histological type, grade, ER, PR, HER2, and Ki67 mitotic index, biological type, radiological status of axillary lymph nodes, lymph node cortical thickness, and ADC values of lymph nodes and tumor in MRI, T and N staging (AJCC-8), SLNB and ALND results, and lymphovascular and perineural invasion status.

The mammography examinations were evaluated for the presence of pathological axillary lymph nodes, tumor size, and mammography BI-RADS results. MRI-assessed parameters included tumor size, pathological axillary lymph node status, axillary pathological lymph node cortical thickness, the number of pathological axillary lymph nodes, minimum, maximum, and average ADC values of axillary lymph nodes, tumor ADC value, and muscle ADC value.

MRI examination was performed in the supine position with a 3T MR device (Siemens Magnetom Skyra, Erlangen, Germany) following the intravenous administration of a contrast agent. ADC maps were generated using three b-values (50, 400, 800 s/mm^2^) in diffusion-weighted imaging. ADC measurements were obtained from ADC maps using the ROI (region of interest). Lymph nodes with a cortical thickness of 3 mm or more on the ipsilateral side of the breast lesion were evaluated, and measurements were made by placing the ROI twice on the lymph node with the thickest cortex on ADC maps. Measurements were recorded as minimum and maximum values using round ROIs drawn to the widest extent without extending beyond the lymph node cortex. The average and the difference between the minimum and maximum ADC values were calculated. Additionally, two measurements were made on the pectoral muscle on the same side as the tumor, and the average values were recorded. Ratios such as lymph node mean ADC, tumor ADC/lymph node mean ADC, muscle ADC, and minimum and maximum values of lymph node ADC were calculated from these measurements. Lymph nodes with a cortical thickness of less than 3 mm were not subjected to diffusion measurement due to the inability to place an adequately sized ROI. Following ADC measurement in MRI, axillary lymph nodes were evaluated by the same radiologist in terms of morphological characteristics (cortical thickness, presence of asymmetric/eccentric thickened cortex, narrowing or absence of hilum, sphericity of the lymph node), and the axillary status in MRI was determined. If there were multiple pathological lymph nodes, the number was noted, and the same lymph node was used for measurements.

SLNB detectiıon was performed with patent blue dye. Blue-stained lymph nodes and all lymph nodes with pathological appearance were excised during the procedure. Axillary lymph node dissection was performed in cases where more than two metastatic lymph nodes were detected in the sentinel lymph node. Postoperative pathology results of all patients were reviewed, and the data were evaluated in our study.

## 3. Statical Analysis

Descriptive statistics for the variables measured in this study were presented in relation to the categorical groups. To determine the distributions of variables according to categorical groups, the Shapiro–Wilk test was applied. Variables indicating normal distribution were analyzed using appropriate normality tests, with results presented as mean and standard deviation. To calculate differences between the groups, *t*-tests and ANOVA tests were employed. For post-hoc comparisons in the ANOVA test, the LSD method was preferred. The Pearson chi-square test was used to identify relationships between categorical variables. Pearson’s correlation coefficient (r) was calculated to identify and interpret relationships between all continuous variables. To assess the consistency of the results for US, MMG, and MRI categorical data, the Kappa test was used, and sensitivity, specificity, and accuracy values were calculated. The receiver operating characteristic (ROC) curve analysis was performed to determine the diagnostic power of various ADC values in identifying the pathological condition. ROC curves were visualized separately for each ADC value, with the area under the curve, cut-off points, and critical value ranges calculated and shared. SPSS version 25 (SPSS Inc., Chicago, IL, USA) was used for data analysis, and a *p*-value of <0.05 was considered statistically significant.

## 4. Results

A total of 104 female participants with a mean age of 55.48 ± 13.93 years were included in our study. Various descriptive statistics related to this study are presented in Table 1 and Table 2.

Ultrasonographic evaluation: The tumor size on ultrasonographic examination ranged from 7 mm to 75 mm, with an average of 24 mm. Among the 40 patients with pathological lymph nodes, the axillary lymph node status was as follows: 1 pathological lymph node in 16 patients, 2 in 10 patients, 3 in 11 patients, 4 in 1 patient, and 5 in 2 patients. The cortical thickness of the pathological lymph nodes in these 40 patients ranged from 3 mm to 20.5 mm, with an average of 6.3 mm.

In mammographic examinations in 28 patients, tumor size was not mentioned because of multicentrity and microcalsification. In 65 patients, tumor size ranged from 4 mm to 76 mm, with an average of 26.43 mm. Pathological axillary lymph nodes were detected in 22 (21.2%) patients, while no pathological lymph nodes were detected in the axilla in 71 (68.3%) patients (Table 1).

As shown in Table 3, the *p*-values in all these comparisons are above the conventional threshold of 0.05, indicating that none of the differences between the “None” and “Present” groups for these MRI variables are statistically significant. This suggests that the pathological condition, as measured by these MRI parameters, does not show a significant association with the observed ADC values in the lymph nodes, tumor, or muscle tissue. There is a marginal difference between tumor/lymph node mean ADC values, but it is not statistically significant.

**Figure 1:**
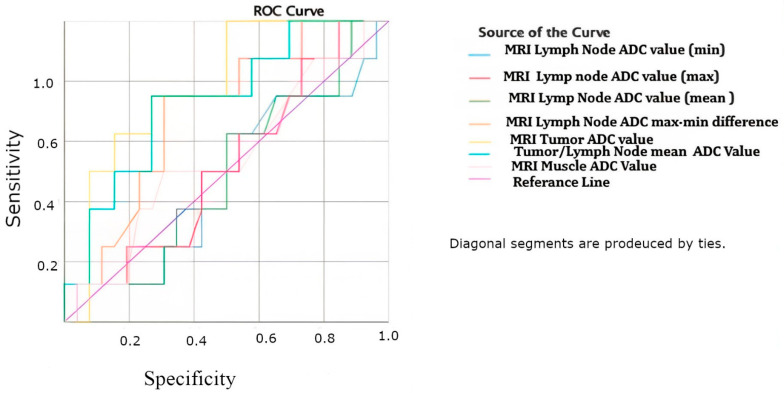
ROC curve analysis for all ADC values.

The diagnostic power, sensitivity, specificity, threshold, and critical values of the ADC values related to the results of the ROC curve analysis are shared in Table 4, and the ROC curves corresponding to these results are shown in Figure 1 below. According to the results, the diagnostic power of the MRI tumor ADC value in detecting the disease was found to be 78.4%, while the diagnostic power of the Tumor ADC/Lymph node mean ADC ratio value was found to be 73.6% and statistically significant in detecting the pathological lymph node (*p* = 0.047). The diagnostic powers of other ADC values were not statistically significant, but the MRI lymph node ADC max–min difference was found to be the result closest to the critical value of *p* < 0.05 (*p* = 0.096). A threshold (cut-off) value of 0.935 with a sensitivity of 0.750 and a specificity of 0.731 was determined for the MRI tumor ADC value. For the Tumor ADC/Lymph node mean ADC ratio, the threshold value corresponding to a sensitivity of 0.750 and a specificity of 0.731 was determined as 1.118.

Although differences are seen in the mean values of subgroups in Table 5, it was seen that there were no statistically significant differences in the mean US tumor sizes, Pathological lymph node numbers, MRI tumor sizes, MRI lymph node cortex thicknesses, MRI tumor ADC values, and MRI lymph node ADC values of the tumor subgroups (*p* = 0.082, 0.457, 0.159, 0.328, 0.479, and 0.263, respectively).

## 5. Discussion

The treatment of breast cancer is surgical. One of the most important prognostic factors in breast cancer is axillary lymph node metastasis. The presence of metastatic axillary lymph nodes is crucial in staging the disease, determining surgical treatment, and guiding medical oncology treatment. Additionally, the 5-year survival rate decreases as the number of metastatic lymph nodes increases [10]. The false-negative rates of SLNB (Sentinel Lymph Node Biopsy) vary between 0% and 29% [11,12]. Since axillary staging is an important parameter in prognosis and treatment, this false negativity is of significant concern. Furthermore, using non-invasive imaging methods to successfully evaluate the axilla preoperatively, instead of the invasive SLNB, could offer benefits such as reduced costs and shorter operation times.

In this study, we compared the preoperative axillary evaluation results obtained from mammography, ultrasonography, and magnetic resonance imaging in breast cancer patients scheduled for surgery in our clinic with the intraoperative SLNB and postoperative axillary intervention pathology results. Our aim was to explore an alternative for axillary interventions by measuring the ADC values of the tumor, lymph nodes, and muscle tissue, seeking reasonable sensitivity and specificity values.

There are multiple studies working on this issue. When studies related to lymph node ADC values are evaluated, in a study by İnanç et al., where they assessed the axillary lymph nodes of 85 breast cancer patients, it was reported that the ADC values of histopathologically confirmed metastatic lymph nodes were 0.89 ± 0.18 × 10^−3^ mm^2^/s, while the values for benign nodes were 1.41 ± 0.21 × 10^−3^ mm^2^/s (*p* < 0.0001). In this study, an ADC cut-off value of 0.985 × 10^−3^ mm^2^/s was selected for distinguishing between benign and malignant nodes, yielding a sensitivity of 83% and a specificity of 98% [13]. Yamaguchi et al., comparing the ADC values of metastatic and non-metastatic axillary lymph nodes, reported the mean ADC values as 0.746 × 10^−3^ mm^2^/s for malignant lymph nodes and 1.034 × 10^−3^ mm^2^/s for benign lymph nodes (*p* < 0.001). In this study, an ADC cut-off value of 0.852 × 10^−3^ mm^2^/s was chosen, resulting in a sensitivity of 85% and a specificity of 91% [14]. In the study by Fornasa et al., the mean ADC value for metastatic lymph nodes was found to be 0.878 × 10^−3^ mm^2^/s, while, for benign lymph nodes, it was 1.494 × 10^−3^ mm^2^/s (*p* < 0.001) [15]. When the ADC cut-off value was set at 1.09 × 10^−3^ mm^2^/s, they found a sensitivity of 94.7%, a specificity of 91.7%, a positive predictive value (PPV) of 90%, and a negative predictive value (NPV) of 95.7% (85). In another study, when the ADC cut-off value was determined as 0.986 × 10^−3^ mm^2^/s, a sensitivity of 75.8% and a specificity of 83.9% were reported [16].

In our study, the mean ADC values for the measured metastatic lymph nodes were 0.820 × 10^−3^ mm^2^/s, while, for benign nodes, the values were 0.850 × 10^−3^ mm^2^/s (Table 4). The cut-off value for the mean ADC was found to be 0.820 × 10^−3^ mm^2^/s, and, when this value was selected, the sensitivity and the specificity of the mean ADC values were determined to be 62% and 50%, respectively (Table 4). Although 3T MRI was used in our study, the mean ADC measurement values for lymph nodes were found to be similar to those in studies conducted with 1.5 T. Consistent with the literature, the mean ADC values for metastatic lymph nodes in our study were found to be lower than those for benign nodes. When sensitivity and specificity are evaluated, our study demonstrates poorer results than other studies. This discrepancy may be due to the fact that measurements were only taken from lymph nodes with a cortex thickness of 3 mm or greater. Zhang et al. have demonstrated that, in the era of artificial intelligence and deep learning, better results are achieved when data are processed through neural networks [17].

In our study, we also found that the tumor ADC value, specifically, the ratio of the tumor ADC value to the lymph node mean ADC value, was more significant for detecting metastasis compared to the axillary lymph node ADC value alone. When the cut-off value for tumor ADC was set at 0.935, the sensitivity was 75%, the specificity was 73%, and the AUC value was 0.784 (*p* = 0.017) (Table 4). The average tumor ADC value in tumors with metastatic lymph nodes was calculated as 0.840 × 10^−3^ mm^2^/s, while, in tumors without lymph node metastasis, the average tumor ADC value was 0.920 × 10^−3^ mm^2^/s (Table 3).

The repeatability of measuring ADC value and inter-reader variability is also debated in the literature. ROI size is significantly correlated with volumetric results. It has been determined that lower histogram percentiles have better repeatability [18]. Mean ADC values of multiple-slice restricted ROI consistency and resulted in similar predictive performance for pathologic complete response between the two readers [19]. In our study, the ROI size used is 3 mm. However, its repeatability has not been investigated.

In a study by You Kim et al., the average ADC value for tumors with metastatic lymph nodes was found to be 0.888 × 10^−3^ mm^2^/s, while, for tumors without lymph node metastasis, the average ADC value was 0.999 × 10^−3^ mm^2^/s. When the cut-off value was set at 0.991 × 10^−3^ mm^2^/s for detecting metastatic lymph nodes, the sensitivity was 86.2%, the specificity was 52.4%, and the AUC value was 0.701 (*p* = 0.001) [20]. Similarly, in the study by Belli et al., the average tumor ADC value in patients with metastatic lymph nodes was 0.980 × 10^−3^ mm^2^/s, compared to 1.080 × 10^−3^ mm^2^/s in patients without metastasis (*p* = 0.001) [21]. Likewise, Cho et al. determined that the tumor ADC value was lower in tumors with lymph node metastasis than in those without, but this finding was statistically non-significant [22].

Our study demonstrated that not only the lymph node ADC value but also the tumor ADC value and the tumor ADC value to lymph node ADC value ratio are important parameters to consider when detecting lymph node metastasis. In our analysis, the tumor ADC value in breast MRI was found to be more significant than the average axillary lymph node ADC value in detecting axillary lymph node metastasis (*p* = 0.017). In cases where there is radiological uncertainty regarding lymph node status, particularly in borderline cases, tumor ADC values can be used to guide clinical decision-making. If the tumor ADC value is below the cut-off value of 0.935 × 10^−3^ mm^2^/s., the possibility of pathological lymph nodes should be considered in practical applications.

In the study by Kim et al., no statistically significant difference was found between tumor ADC values and tumor subtypes (*p* = 0.051) [20]. When evaluating the ADC values of tumor subtypes with different prognostic characteristics, we did not find a statistically significant difference (*p* = 0.479), which is consistent with the literature.

## 6. Conclusions and Recommendations

In our current research, breast MRI was found to be the most effective pre-operative, non-invasive method for determining the status of axillary lymph nodes. Contrary to what was initially expected, the ADC value of axillary lymph nodes showed lower sensitivity and specificity rates in evaluating lymph node metastasis compared to the tumor ADC value. However, measurements of the tumor ADC value and the tumor ADC/lymph node average ADC ratio appear to be the parameters most closely associated with indicating metastatic lymph nodes. Reaching these conclusions with this sample size may, of course, invite skepticism. However, more definitive results can be obtained with a larger number of patients. The data obtained in our study suggest that, under current conditions, there is no radiological imaging technique with sufficient sensitivity or specificity to replace SLNB (Sentinel Lymph Node Biopsy) in detecting axillary lymph node metastasis.

## Figures and Tables

**Table 1:** Descriptive statistics of surgery and imaging modalities.

Variable	Category	Frequency (%)
Surgeries Performed	Modified Radical Mastectomy	32 (30.8)
	BCS+SLNB	28 (26.9)
	BCS+ALND	8 (7.7)
	Mastectomy+SLNB	36 (34.6)
Tumor Location	Left	59 (56.7)
	Right	45 (43.3)
Multicentricity/Multifocality	None	87 (83.7)
	Present	17 (16.3)
US Axilla Pathological Lymph Node Status	None	64 (61.5)
	Present	40 (38.4))
US Birads	4	50 (48)
	5	46 (44.2)
	6	8 (7.6)
MMG Axilla Pathological Lymph Node Status	None	71 (68.3)
	Present	22 (21.2)
MMG Birads	4	29 (27.9)
	5	30 (28.8)
	6	8 (7.7)
Mri Axilla Pathological Lymph Node Status	None	60 (57.6)
	Present	44 (42.3)

**Table 2:** Descriptive statistics of stage and pathologic evaluation.

Variable	Category	Frequency (%)
Tumor Histopathological Type	Invasive Ductal Carcinoma	94 (90.4)
	Invasive Ductal Carcinoma	5 (4.8)
	Ductal Carcinoma In Situ	5 (4.8)
Tumor Subgroups	Luminal A	41 (39.4)
	Luminal B	42 (40.3)
	Her 2 (+)	5 (4.8)
	Triple (−)	16 (15.3)
		
Pt Stage	T Is	22 (21.1)
	T 1	1 (1.0)
	T 1a	1 (1.0)
	T 1b	10 (9.6)
	T 1c	52 (50)
	T 2	11 (10.6)
	T 3	1 (1.0)
	T 4	2 (1.9)
	T 4b	4 (3.8)
		
Pn Stage	N 0	63 (60.5)
	N 1a	20 (19.2)
	N 1b	1 (1.0)
	N 2a	9 (8.7)
	N 3a	11 (10.6)
Pathological Axillary Metastasis Status	None	64 (61.5)
	Present	40 (38.5)
Lymphovascular Invasion	None	53 (50.9)
	Present	51 (49.1)
Perineural Invasion	None	67 (64.4)
	Present	37 (35.5)

**Table 3:** MRI-related ADC measurements comparing two groups: those with the presence of a pathological condition (denoted as “Present”) and those without it (denoted as “None”). The *p*-value was obtained based on the *t*-test result.

Variables	N (Pathological)	Mean	Standard Deviation	*p*
MRI Lymph node ADC value min.	None Present	0.77 0.76	0.15 0.13	0.880
MRI Lymph node ADC Value Max.	None Present	0.94 0.88	0.20 0.14	0.329
MRI Lymph node ADC Value mean	None Present	0.85 0.82	0.17 0.13	0.542
MRI Lymph Node ADC Value Max–Min Difference	None Present	0.17 0.12	0.10 0.08	0.120
MRI Tumor ADC Value	None Present	0.92 0.84	0.27 0.22	0.162
Tumor/Lymph Node Mean Adc	None Present	1.26 1.05	0.34 0.28	0.078
MRI Muscle ADC Value	None Present	1.00 1.03	0.13 0.13	0.275

**Table 4:** Detection power, sensitivity, specificity, threshold, and critical values of ADC values in detecting the disease condition identified by pathology results.

Parameter	95% CI	*p*	Sensitivity	Specificity	Cut-Off	Critical Value
MRI Tumor ADC value	0.784	0.017	0.750	0.731	0.935	0.810–1.055
Tumor ADC/Lymph Node mean value	0.736	0.047	0.750	0.731	1.118	0.843–1.317
MRI Lymph Node ADC max–min difference	0.697	0.096	0.750	0.692	0.150	0.065–0.330
MRI Muscle ADC value	0.546	0.700	0.500	0.538	1.055	0.845–1.160
MRI Lymph Node ADC max value	0.524	0.839	0.625	0.462	0.855	0.720–1.090
MRI Lymph Node ADC min value	0.474	0.823	0.625	0.462	0.725	0.525–0.945
MRI Lymph Node ADC mean value	0.498	0.984	0.625	0.500	0.820	0.625–1.005

ROC: Receiver operating characteristics.

**Table 5:** Effects of tumor subgroups on various variables and *p*-value obtained from ANOVA test result.

Variable	Tumor Subgroup	Mean (mm)	Standard Deviation	*p*-Value
USG Tumor Size	Luminal A	20.61	14.08	0.082
	Luminal B	25.60	12.54	
	HER 2	23.20	4.44	
	TRIPLE (−)	31.13	17.05	
MRI Tumor Size	Luminal A	24.18	17.35	0.159
	Luminal B	30.61	16.93	
	HER 2	25.00	3.46	
	TRIPLE (−)	37.00	23.25	
MRI Lymph Node Cortex Thickness	Luminal A	5.96	1.81	0.328
	Luminal B	8.66	5.42	
	HER 2	11.00	n/a	
	TRIPLE (−)	9.30	3.85	
MRI Tumor ADC Value	Luminal Luminal A	0.87	0.27	0.479
	B	0.85	0.21	
	HER 2	1.03	0.14	
	TRIPLE (−)	0.92	0.24	
MRI Lymph Node ADC Value (Mean)	Luminal A	0.86	0.17	0.263
	Luminal B	0.83	0.12	
	HER 2	0.92	n/a	
	TRIPLE (−)	0.73	0.14	

## Data Availability

Due to ethical concerns, the study data cannot be disclosed publicly. However, it can be provided to the editor or reviewers upon request.

## References

[1] Siegel R., Naishadham D., Jemal A. (2012). Cancer statistics, 2012. CA Cancer J. Clin..

[2] Woelfel I.A., Fernandez L.J., Idowu M.O., Takabe K. (2018). A high burden of comorbid conditions leads to decreased survival in breast cancer. Gland. Surg..

[3] Samiei S., Simons J.M., Engelen S.M.E., Beets-Tan R.G.H., Classe J.M., Smidt M.L. (2021). Axillary Pathologic Complete Response After Neoadjuvant Systemic Therapy by Breast Cancer Subtype in Patients with Initially Clinically Node-Positive Disease: A Systematic Review and Meta-analysis. JAMA Surg..

[4] Blanchard D.K., Donohue J.H., Reynolds C., Grant C.S., McMasters K., Velasco J.M. (2003). Relapse and morbidity in patients undergoing sentinel lymph node biopsy alone or with axillary dissection for breast cancer. Arch. Surg..

[5] Gillespie T.C., Sayegh H.E., Brunelle C.L., Daniell K.M., Taghian A.G. (2018). Breast cancer-related lymphedema: Risk factors, precautionary measures, and treatments. Gland. Surg..

[6] Ahn H.S., Jang M., Kim S.M., La Y.B., Lee S.H. (2019). Usefulness of preoperative breast magnetic resonance imaging with a dedicated axillary sequence for the detection of axillary lymph node metastasis in patients with early ductal breast cancer. Radiol. Medica.

[7] Liu G., Zhang M.K., He Y., Li X.R., Wang Z.L. (2019). Shear wave elasticity of breast lesions: Would it be correlated with the extracellular matrix components?. Gland. Surg..

[8] Liu G., Zhang M.K., He Y., Liu Y., Li X.R., Wang Z.L. (2019). BI-RADS 4 breast lesions: Could multi-mode ultrasound be helpful for their diagnosis?. Gland. Surg..

[9] Gentilini O.D., Botteri E., Sangalli C., Galimberti V., Porpiglia M., Agresti R., Luini A., Viale G., Cassano E., Peradze N. (2023). Sentinel Lymph Node Biopsy vs No Axillary Surgery in Patients with Small Breast Cancer and Negative Results on Ultrasonography of Axillary Lymph Nodes: The SOUND Randomized Clinical Trial. JAMA Oncol..

[10] Fisher B., Bauer M., Lawrence Wickerham D., Redmond C.K., Fisher E.R., Cruz A.B., Foster R., Gardner B., Lerner H., Margolese R. (1983). Relation of Number of Positive Axillary Nodes to the Prognosis of Patients with Primary Breast Cancer. An NSABP Update. Cancer.

[11] Liu Y., Wang Y., Feng S., Xu Z., Yao M., Huang X., Li P., Wu X., Liu C., Chen X. (2023). Axillary ultrasound after neoadjuvant therapy reduces the false-negative rate of sentinel lymph node biopsy in patients with cytologically node-positive breast cancer. Breast Cancer Res. Treat..

[12] Rozenberg S., Liebens F., Ham H. (1999). The sentinel node in breast cancer: Acceptable false-negative rate. Lancet.

[13] Guvenc I., Whitman G.J., Liu P., Yalniz C., Ma J., Dogan B.E. (2019). Diffusion-weighted MR imaging increases diagnostic accuracy of breast MR imaging for predicting axillary metastases in breast cancer patients. Breast J..

[14] Yamaguchi K., Schacht D., Nakazono T., Irie H., Abe H. (2015). Diffusion weighted images of metastatic as compared with nonmetastatic axillary lymph nodes in patients with newly diagnosed breast cancer. J. Magn. Reson. Imaging.

[15] Fornasa F., Nesoti M.V., Bovo C., Bonavina M.G. (2012). Diffusion-weighted magnetic resonance imaging in the characterization of axillary lymph nodes in patients with breast cancer. J. Magn. Reson. Imaging.

[16] Kim E.J., Kim S.H., Kang B.J., Choi B.G., Song B.J., Choi J.J. (2014). Diagnostic value of breast MRI for predicting metastatic axillary lymph nodes in breast cancer patients: Diffusion-weighted MRI and conventional MRI. Magn. Reson. Imaging..

[17] Zhang X., Liu M., Ren W., Sun J., Wang K., Xi X., Zhang G. (2022). Predicting of axillary lymph node metastasis in invasive breast cancer using multiparametric MRI dataset based on CNN model. Front. Oncol..

[18] Newitt D.C., Amouzandeh G., Partridge S.C., Marques H.S., Herman B.A., Ross B.D., Hylton N.M., Chenevert T.L., Malyarenko D.I. (2020). Repeatability and Reproducibility of ADC Histogram Metrics from the ACRIN 6698 Breast Cancer Therapy Response Trial. Tomogr.

[19] Le N.N., Li W., Onishi N., Newitt D.C., Gibbs J.E., Wilmes L.J., Kornak J., Partridge S.C., LeStage B., Price E.R. (2022). Effect of Inter-Reader Variability on Diffusion-Weighted MRI Apparent Diffusion Coefficient Measurements and Prediction of Pathologic Complete Response for Breast Cancer. Tomography.

[20] Kim J.Y., Seo H.B., Park S., Moon J., Il, Lee J.W., Lee N.K., Lee S.W., Bae Y.T. (2015). Early-stage invasive ductal carcinoma: Association of tumor apparent diffusion coefficient values with axillary lymph node metastasis. Eur. J. Radiol..

[21] Belli P., Costantini M., Bufi E., Giardina G.G., Rinaldi P., Franceschini G., Bonomo L. (2015). Diffusion magnetic resonance imaging in breast cancer characterisation: Correlations between the apparent diffusion coefficient and major prognostic factors. Radiol. Med..

[22] Cho P., Park C.S., Park G.E., Kim S.H., Kim H.S., Oh S.J. (2023). Diagnostic Usefulness of Diffusion-Weighted MRI for Axillary Lymph Node Evaluation in Patients with Breast Cancer. Diagnostics.

